# A Hilbert-type integral inequality in the whole plane related to the kernel of exponent function

**DOI:** 10.1186/s13660-018-1834-4

**Published:** 2018-09-12

**Authors:** Yanru Zhong, Meifa Huang, Bicheng Yang

**Affiliations:** 1 School of Computer Science and Information Security, Guilin University of Electronic Technology, Guilin, P.R. China; 2 School of Mechanical Science and Electrical Engineering, Guilin University of Electronic Technology, Guilin, P.R. China; 3 Department of Mathematics, Guangdong University of Education, Guangzhou, P.R. China

**Keywords:** 26D15, 31A10, Hilbert-type integral inequality, Weight function, Intermediate variable, Equivalent statement, Operator, Gamma function

## Abstract

By using real analysis and weight functions, we obtain a few equivalent statements of a Hilbert-type integral inequality in the whole plane related to the kernel of exponent function with intermediate variables. The constant factor related to the gamma function is proved to be the best possible. We also consider some particular cases and the operator expressions.

## Introduction

If $0 < \int_{0}^{\infty} f^{2}(x)\,dx < \infty $ and $0 < \int_{0}^{\infty} g^{2}(y)\,dy < \infty $, then we have the following well-known Hilbert integral inequality (see [1]):

1 $$ \int_{0}^{\infty} \int_{0}^{\infty} \frac{f(x)g(y)}{x + y}\,dx \,dy < \pi \biggl( \int_{0}^{\infty} f^{2}(x)\,dx \int_{0}^{\infty} g^{2}(y)\,dy \biggr)^{\frac{1}{2}}, $$

where the constant factor *π* is the best possible. In 1925, by introducing the pair of conjugate exponents $(p,q)$ ($p > 1$, $\frac{1}{p} + \frac{1}{q} = 1 $), Hardy et al. gave an extension of (1) (see [1], Theorem 316). Recently, by means of weight functions, some new extensions of (1) and the Hardy’s work were given by Yang [2, 3] and in [4–9]. Most of them are built in the quarter plane of the first quadrant.

In 2007, Yang [10] provided a Hilbert-type integral inequality in the whole plane with the exponent function and intermediate variables as follows:

2 $$ \int_{ - \infty}^{\infty} \int_{ - \infty}^{\infty} \frac{f(x)g(y)}{(1 + e^{x + y})^{\lambda}} \,dx \,dy < B\biggl( \frac{\lambda}{2},\frac{\lambda}{2}\biggr) \biggl( \int_{ - \infty}^{\infty} e^{ - \lambda x}f^{2}(x)\,dx \int_{ - \infty}^{\infty} e^{ - \lambda y}g^{2}(y)\,dy \biggr)^{\frac{1}{2}}, $$

where the constant factor $B(\frac{\lambda}{2},\frac{\lambda}{2}) $ is the best possible ($\lambda > 0$, $B(u,v) $ is the beta function). He et al. [11–19] proved some new Hilbert-type integral inequalities in the whole plane with the best possible constant factors.

In 2017, Hong [20] gave two equivalent statements between Hilbert-type inequalities with general homogenous kernel and a few parameters. A few authors continue to study this topic (see [21–25]).

In this paper, by using real analysis and weight functions we obtain a few equivalent statements of a Hilbert-type integral inequality in the whole plane related to the exponent function with intermediate variables. The constant factor related to the gamma function is proved to be the best possible. We also consider some particular cases and operator expressions.

## Some lemmas

For $\gamma,\rho,\sigma > 0 $, setting $h(u): = e^{ - \rho u^{\gamma}}$ ($u > 0 $), we find

3 $$ \begin{aligned}[b] k_{\rho}^{(\gamma )}(\sigma )&: = \int_{0}^{\infty} h(u)u^{\sigma - 1}\,du = \int_{0}^{\infty} e^{ - \rho u^{\gamma}} u^{\sigma - 1}\,du {} \quad \bigl(v = \rho u^{\gamma} \bigr)\\ &= \frac{1}{\gamma \rho^{\sigma /\gamma}} \int_{0}^{\infty} e^{ - v}v^{(\sigma /\gamma ) - 1}\,dv = \frac{\Gamma (\sigma /\gamma )}{\gamma \rho^{\sigma /\gamma}} \in \mathbb{R}_{ +} = (0,\infty ), \end{aligned} $$

where $\Gamma (s): = \int_{0}^{\infty} e^{ - v}v^{s - 1}\,dv$ ($\operatorname{Re} s > 0 $) is the gamma function (see [26]).

For $\delta \in \{ - 1,1\}$, $\alpha,\beta \in ( - 1,1) $, we set

$$\begin{gathered} x_{\alpha}: = \vert x \vert + \alpha x,\qquad y_{\beta}: = \vert y \vert + \beta y\quad \bigl(x,y \in \mathbb{R} = ( - \infty,\infty )\bigr), \\ E_{\delta}: = \bigl\{ t \in \mathbb{R}; \vert t \vert ^{\delta} \ge 1\bigr\} ,\qquad E_{ - \delta} = \bigl\{ t \in \mathbb{R}; \vert t \vert ^{\delta} \le 1\bigr\} . \end{gathered} $$

### Lemma 1

*For* $c > 0$, $\theta = \alpha, \beta \in ( - 1,1) $, *we have*

4 $$\begin{aligned}& \int_{E_{\delta}} t_{\theta}^{ - c\delta - 1} \,dt = \frac{1}{c} \biggl[ \frac{1}{(1 + \theta )^{c\delta + 1}} + \frac{1}{(1 - \theta )^{c\delta + 1}} \biggr], \end{aligned}$$

5 $$\begin{aligned}& \int_{E_{ - \delta}} t_{\theta}^{c\delta - 1} \,dt = \frac{1}{c} \biggl[ \frac{1}{(1 + \theta )^{ - c\delta + 1}} + \frac{1}{(1 - \theta )^{ - c\delta + 1}} \biggr]; \end{aligned}$$

*and for* $c \le 0 $, *we have*

$$\int_{E_{\delta}} t_{\theta}^{ - c\delta - 1} \,dt = \int_{E_{ - \delta}} t_{\theta}^{c\delta - 1} \,dt = \infty. $$

### Proof

Setting $E_{\delta}^{ +}: = \{ t \in \mathbb{R}_{ +};t^{\delta} \ge 1\}$, $E_{\delta}^{ -}: = \{ - t \in \mathbb{R}_{ +};( - t)^{\delta} \ge 1\} $, we find $E_{\delta} = E_{\delta}^{ +} \cup E_{\delta}^{ -} $ and

$$\begin{aligned} \int_{E_{\delta}} t_{\theta}^{ - c\delta - 1} \,dt& = \int_{E_{\delta}^{ +}} \bigl[(1 + \theta )t\bigr]^{ - c\delta - 1}\,dt + \int_{E_{\delta}^{ -}} \bigl[(1 - \theta ) ( - t)\bigr]^{ - c\delta - 1}\,dt \\ &= \biggl[ \frac{1}{(1 + \theta )^{c\delta + 1}} + \frac{1}{(1 - \theta )^{c\delta + 1}} \biggr] \int_{E_{\delta}^{ +}} t^{ - c\delta - 1}\,dt. \end{aligned} $$

Setting $t = u^{\frac{1}{\delta}} $, we find

$$\int_{E_{\delta}^{ +}} t^{ - c\delta - 1}\,dt = \frac{1}{ \vert \delta \vert } \int_{1}^{\infty} u^{\frac{1}{\delta} ( - c\delta - 1)} u^{\frac{1}{\delta} - 1}\,du = \int_{1}^{\infty} u^{ - c - 1} \,du. $$

Hence, for $c > 0 $, (4) follows, and for $c \le 0$, $\int_{E_{\delta}} t_{\theta}^{ - c\delta - 1} \,dt = \infty $. Since, for $c > 0 $,

$$\int_{E_{ - \delta}} t_{\theta}^{c\delta - 1} \,dt = \int_{E_{( - \delta} )} t_{\theta}^{ - c( - \delta ) - 1} \,dt = \biggl[ \frac{1}{(1 + \theta )^{ - c\delta + 1}} + \frac{1}{(1 - \theta )^{ - c\delta + 1}} \biggr] \int_{0}^{1} u^{c - 1} \,du, $$

we have (5), and for $c \le 0$, $\int_{E_{ - \delta}} t_{\theta}^{c\delta - 1} \,dt = \infty $.

The lemma is proved. □

In the following, We further assume that $p > 1$, $\frac{1}{p} + \frac{1}{q} = 1$, $\delta \in \{ - 1,1\}$, $\alpha,\beta \in ( - 1,1)$, $\gamma,\rho,\sigma > 0$, $\sigma_{1} \in \mathbb{R}$, $k_{\rho}^{(\gamma )}(\sigma ) $ is given by (3), and

6 $$ K_{\alpha,\beta}^{(\gamma )}(\sigma ): = \frac{2k_{\rho}^{(\gamma )}(\sigma )}{(1 - \alpha^{2})^{1/q}(1 - \beta^{2})^{1/p}}. $$

For $n \in \mathbb{N} = \{ 1,2, \ldots \}$, $E_{ - 1} = [ - 1,1]$, $x \in E_{\delta} $, we define:

$$\begin{gathered} I^{( - )}(x): = \int_{ - 1}^{0} e^{ - \rho (x_{\alpha}^{\delta} y_{\beta} )^{\gamma}} y_{\beta}^{\sigma + \frac{1}{qn} - 1} \,dy,\qquad I^{( + )}(x): = \int_{0}^{1} e^{ - \rho (x_{\alpha}^{\delta} y_{\beta} )^{\gamma}} y_{\beta}^{\sigma + \frac{1}{qn} - 1} \,dy, \\ I(x): = I^{( - )}(x) + I^{( + )}(x) = \int_{E_{ - 1}} e^{ - \rho (x_{\alpha}^{\delta} y_{\beta} )^{\gamma}} y_{\beta}^{\sigma + \frac{1}{qn} - 1} \,dy. \end{gathered} $$

For $y_{\beta} = (\operatorname{sgn} (y) + \beta )y $, where

$$\begin{gathered} \operatorname{sgn} (y): = \textstyle\begin{cases} - 1,&y < 0, \\ 0,&y = 0, \\ 1,&y > 0, \end{cases}\displaystyle \\ x_{\alpha}^{\delta} = \bigl(1 + \alpha \operatorname{sgn} (x) \bigr)^{\delta} \vert x \vert ^{\delta} \ge \min _{\delta \in \{ - 1,1\}} \bigl\{ \bigl(1 \pm \vert \alpha \vert \bigr)^{\delta} \bigr\} \quad (x \in E_{\delta} ), \end{gathered} $$

and $1 - |\alpha | \le (1 + |\alpha |)^{ - 1} \le 1 + |\alpha | \le (1 - |\alpha |)^{ - 1} $, we have

7 $$ (1 \pm \beta )x_{\alpha}^{\delta} \ge m_{\alpha,\beta}: = \bigl(1 - \vert \beta \vert \bigr) \bigl(1 - \vert \alpha \vert \bigr) > 0\quad (x \in E_{\delta} ). $$

For fixed $x \in E_{\delta} $, setting $u = x_{\alpha}^{\delta} y_{\beta} $, we find

8 $$\begin{aligned}& I^{( - )}(x) = \frac{x_{\alpha}^{ - \delta (\sigma + \frac{1}{qn})}}{1 - \beta} \int_{0}^{(1 - \beta )x_{\alpha}^{\delta}} e^{ - \rho u^{\gamma}} u^{\sigma + \frac{1}{qn} - 1}\,du \ge \frac{x_{\alpha}^{ - \delta (\sigma + \frac{1}{qn})}}{1 - \beta} \int_{0}^{m_{\alpha,\beta}} e^{ - \rho u^{\gamma}} u^{\sigma + \frac{1}{qn} - 1}\,du,\hspace{-20pt} \\& I^{( + )}(x) = \frac{x_{\alpha}^{ - \delta (\sigma + \frac{1}{qn})}}{1 + \beta} \int_{0}^{(1 + \beta )x_{\alpha}^{\delta}} e^{ - \rho u^{\gamma}} u^{\sigma + \frac{1}{qn} - 1}\,du \ge \frac{x_{\alpha}^{ - \delta (\sigma + \frac{1}{qn})}}{1 + \beta} \int_{0}^{m_{\alpha,\beta}} e^{ - \rho u^{\gamma}} u^{\sigma + \frac{1}{qn} - 1}\,du,\hspace{-20pt} \\& \begin{aligned}[b] I(x) &= x_{\alpha}^{ - \delta (\sigma + \frac{1}{qn})} \biggl[ \frac{1}{1 - \beta} \int_{0}^{(1 - \beta )x_{\alpha}^{\delta}} e^{ - \rho u^{\gamma}} u^{\sigma + \frac{1}{qn} - 1}\,du + \frac{1}{1 + \beta} \int_{0}^{(1 + \beta )x_{\alpha}^{\delta}} e^{ - \rho u^{\gamma}} u^{\sigma + \frac{1}{qn} - 1}\,du \biggr]\hspace{-20pt} \\ &\ge \frac{2x_{\alpha}^{ - \delta (\sigma + \frac{1}{qn})}}{1 + \beta} \int_{0}^{m_{\alpha,\beta}} e^{ - \rho u^{\gamma}} u^{\sigma + \frac{1}{qn} - 1}\,du. \end{aligned} \end{aligned}$$

For $n \in \mathbb{N} = \{ 1,2, \ldots \}$, $x \in E_{ - \delta} $, we define:

$$\begin{gathered} J^{( - )}(x): = \int_{ - \infty}^{ - 1} e^{ - \rho (x_{\alpha}^{\delta} y_{\beta} )^{\gamma}} y_{\beta}^{\sigma - \frac{1}{qn} - 1} \,dy,\\ J^{( + )}(x): = \int_{1}^{\infty} e^{ - \rho (x_{\alpha}^{\delta} y_{\beta} )^{\gamma}} y_{\beta}^{\sigma - \frac{1}{qn} - 1} \,dy, \\ J(x): = J^{( - )}(x) + J^{( + )}(x) = \int_{E_{ - 1}} e^{ - \rho (x_{\alpha}^{\delta} y_{\beta} )^{\gamma}} y_{\beta}^{\sigma - \frac{1}{qn} - 1} \,dy. \end{gathered} $$

Since, for $x \in E_{ - \delta} $,

$$x_{\alpha}^{\delta} = \bigl(1 + \alpha \operatorname{sgn} (x) \bigr)^{\delta} \vert x \vert ^{\delta} \le \max _{\delta \in \{ - 1,1\}} \bigl\{ \bigl(1 \pm \vert \alpha \vert \bigr)^{\delta} \bigr\} = \bigl(1 - \vert \alpha \vert \bigr)^{ - 1}, $$

we have

9 $$ M_{\alpha,\beta}: = \bigl(1 + \vert \beta \vert \bigr) \bigl(1 - \vert \alpha \vert \bigr)^{ - 1} \ge \bigl(1 \pm \vert \beta \vert \bigr)x_{\alpha}^{\delta}\quad (x \in E_{ - \delta} ). $$

For fixed $x \in E_{ - \delta} $, setting $u = x_{\alpha}^{\delta} y_{\beta} $, we find

10 $$\begin{aligned}& J^{( - )}(x) = \frac{x_{\alpha}^{ - \delta (\sigma - \frac{1}{qn})}}{1 - \beta} \int_{(1 - \beta )x_{\alpha}^{\delta}}^{\infty} e^{ - \rho u^{\gamma}} u^{\sigma - \frac{1}{qn} - 1}\,du \ge \frac{x_{\alpha}^{ - \delta (\sigma - \frac{1}{qn})}}{1 - \beta} \int_{M_{\alpha,\beta}}^{\infty} e^{ - \rho u^{\gamma}} u^{\sigma - \frac{1}{qn} - 1}\,du,\hspace{-20pt} \\& J^{( + )}(x) = \frac{x_{\alpha}^{ - \delta (\sigma - \frac{1}{qn})}}{1 + \beta} \int_{(1 + \beta )x_{\alpha}^{\delta}}^{\infty} e^{ - \rho u^{\gamma}} u^{\sigma - \frac{1}{qn} - 1}\,du \ge \frac{x_{\alpha}^{ - \delta (\sigma - \frac{1}{qn})}}{1 + \beta} \int_{M_{\alpha,\beta}}^{\infty} e^{ - \rho u^{\gamma}} u^{\sigma - \frac{1}{qn} - 1}\,du,\hspace{-20pt} \\& \begin{aligned}[b] J(x) &= x_{\alpha}^{ - \delta (\sigma - \frac{1}{qn})} \biggl[ \frac{1}{1 - \beta} \int_{(1 - \beta )x_{\alpha}^{\delta}}^{\infty} e^{ - \rho u^{\gamma}} u^{\sigma - \frac{1}{qn} - 1}\,du + \frac{1}{1 + \beta} \int_{(1 + \beta )x_{\alpha}^{\delta}}^{\infty} e^{ - \rho u^{\gamma}} u^{\sigma - \frac{1}{qn} - 1}\,du \biggr]\hspace{-20pt} \\ &\ge \frac{2x_{\alpha}^{ - \delta (\sigma - \frac{1}{qn})}}{1 + \beta} \int_{M_{\alpha,\beta}}^{\infty} e^{ - \rho u^{\gamma}} u^{\sigma - \frac{1}{qn} - 1}\,du. \end{aligned} \end{aligned}$$

In view of (8) and (10), we have the following:

### Lemma 2

*We have the following inequalities*:

11 $$\begin{aligned}& \begin{aligned}[b] I_{1}&: = \int_{E_{\delta}} I(x)x_{\alpha}^{\delta (\sigma_{1} - \frac{1}{pn}) - 1}\,dx \\ &\ge \frac{2}{1 - \beta^{2}} \int_{E_{\delta}} x_{\alpha}^{ - \delta (\sigma - \sigma_{1} + \frac{1}{n}) - 1}\,dx \int_{0}^{m_{\alpha,\beta}} e^{ - \rho u^{\gamma}} u^{\sigma + \frac{1}{qn} - 1}\,du, \end{aligned} \end{aligned}$$

12 $$\begin{aligned}& \begin{aligned}[b] J_{1}&: = \int_{E_{ - \delta}} J(x)x_{\alpha}^{\delta (\sigma_{1} + \frac{1}{pn}) - 1}\,dx \\ &\ge \frac{2}{1 - \beta^{2}} \int_{E_{ - \delta}} x_{\alpha}^{\delta (\sigma_{1} - \sigma + \frac{1}{n}) - 1}\,dx \int_{M_{\alpha,\beta}}^{\infty} e^{ - \rho u^{\gamma}} u^{\sigma - \frac{1}{qn} - 1}\,du. \end{aligned} \end{aligned}$$

### Lemma 3

*If there exists a constant* *M* *such that*, *for any nonnegative measurable functions* $f(x) $ *and* $g(y) $ *in* $\mathbb{R} $,

13 $$ \begin{aligned}[b] I&: = \int_{ - \infty}^{\infty} \int_{ - \infty}^{\infty} e^{ - \rho (x_{\alpha}^{\delta} y_{\beta} )^{\gamma}} f(x)g(y)\,dx \,dy \\ &\le M \biggl[ \int_{ - \infty}^{\infty} x_{\alpha}^{p(1 - \delta \sigma_{1}) - 1}f^{p}(x) \,dx \biggr]^{\frac{1}{p}} \biggl[ \int_{ - \infty}^{\infty} y_{\beta}^{q(1 - \sigma ) - 1}g^{q}(y) \,dy \biggr]^{\frac{1}{q}}, \end{aligned} $$

*then we have* $\sigma_{1} = \sigma $.

### Proof

If $\sigma_{1} > \sigma $, then for $n \ge \frac{1}{\sigma_{1} - \sigma} $ ($n \in \mathbb{N}$), we define the functions:

$$f_{n}(x): = \textstyle\begin{cases} x_{\alpha}^{\delta (\sigma_{1} - \frac{1}{pn}) - 1},&x \in E_{\delta}, \\ 0,&x \in \mathbb{R}\backslash E_{\delta}, \end{cases}\displaystyle \qquad g_{n}(y): = \textstyle\begin{cases} y_{\beta}^{\sigma + \frac{1}{qn} - 1},&y \in E_{ - 1}, \\ 0,&y \in \mathbb{R}\backslash E_{ - 1}, \end{cases} $$

and by (4) and (5) it follows that

$$\begin{aligned} \tilde{J}_{1}&: = \biggl[ \int_{ - \infty}^{\infty} x_{\alpha}^{p(1 - \delta \sigma_{1}) - 1}f_{n}^{p}(x) \,dx \biggr]^{\frac{1}{p}} \biggl[ \int_{ - \infty}^{\infty} y_{\beta}^{q(1 - \sigma ) - 1}g_{n}^{q}(y) \,dy \biggr]^{\frac{1}{q}} \\ &= \biggl( \int_{E_{\delta}} x_{\alpha}^{ - \frac{\delta}{n} - 1}\,dx \biggr)^{\frac{1}{p}} \biggl( \int_{E_{ - 1}} y_{\beta}^{\frac{1}{n} - 1}\,dy \biggr)^{\frac{1}{q}} \\ &= n \biggl[ \frac{1}{(1 + \alpha )^{\frac{\delta}{n} + 1}} + \frac{1}{(1 - \alpha )^{\frac{\delta}{n} + 1}} \biggr]^{\frac{1}{p}} \biggl[ \frac{1}{(1 + \beta )^{\frac{ - 1}{n} + 1}} + \frac{1}{(1 - \beta )^{\frac{ - 1}{n} + 1}} \biggr]^{\frac{1}{q}} < \infty. \end{aligned} $$

By (11) and (13) (for $f = f_{n}$, $g = g_{n} $) we have

$$\begin{gathered} \frac{2}{1 - \beta^{2}} \int_{E_{\delta}} x_{\alpha}^{ - \delta (\sigma - \sigma_{1} + \frac{1}{n}) - 1}\,dx \int_{0}^{m_{\alpha,\beta}} e^{ - \rho u^{\gamma}} u^{\sigma + \frac{1}{qn} - 1}\,du \\ \quad \le I_{1} = \int_{ - \infty}^{\infty} \int_{ - \infty}^{\infty} e^{ - \rho (x_{\alpha}^{\delta} y_{\beta} )^{\gamma}} f_{n}(x)g_{n}(y) \,dx \,dy \le M\tilde{J}_{1} < \infty. \end{gathered} $$

Since for any $n \ge \frac{1}{\sigma_{1} - \sigma}$, $\sigma - \sigma_{1} + \frac{1}{n} \le 0 $, by Lemma 1 it follows that $\int_{E_{\delta}} x_{\alpha}^{ - \delta (\sigma - \sigma_{1} + \frac{1}{n}) - 1}\,dx = \infty $. In view of $\int_{0}^{m_{\alpha,\beta}} e^{ - \rho u^{\gamma}} u^{\sigma + \frac{1}{qn} - 1}\,du > 0 $, we find that $\infty \le M\tilde{J}_{1} < \infty $, which is a contradiction.

If $\sigma_{1} < \sigma $, then for $n \ge \frac{1}{\sigma - \sigma_{1}}$ ($n \in \mathbb{N} $), we define the functions:

$$\tilde{f}_{n}(x): = \textstyle\begin{cases} x_{\alpha}^{\delta (\sigma + \frac{1}{pn}) - 1},&x \in E_{ - \delta}, \\ 0,&x \in \mathbb{R}\backslash E_{ - \delta}, \end{cases}\displaystyle \qquad \tilde{g}_{n}(y): = \textstyle\begin{cases} y_{\beta}^{\sigma - \frac{1}{qn} - 1},&y \in E_{1}, \\ 0,&y \in \mathbb{R}\backslash E_{1}, \end{cases} $$

and by (4) and (5) it follows that

$$\begin{aligned} \tilde{J}_{2}&: = \biggl[ \int_{ - \infty}^{\infty} x_{\alpha}^{p(1 - \delta \sigma_{1}) - 1} \tilde{f}_{n}^{p}(x)\,dx \biggr]^{\frac{1}{p}} \biggl[ \int_{ - \infty}^{\infty} y_{\beta}^{q(1 - \sigma ) - 1} \tilde{g}_{n}^{q}(y)\,dy \biggr]^{\frac{1}{q}} \\ &= \biggl( \int_{E_{ - \delta}} x_{\alpha}^{\frac{\delta}{n} - 1}\,dx \biggr)^{\frac{1}{p}} \biggl( \int_{E_{1}} y_{\beta}^{\frac{ - 1}{n} - 1}\,dy \biggr)^{\frac{1}{q}} \\ &= n \biggl[ \frac{1}{(1 + \alpha )^{\frac{ - \delta}{n} + 1}} + \frac{1}{(1 - \alpha )^{\frac{ - \delta}{n} + 1}} \biggr]^{\frac{1}{p}} \biggl[ \frac{1}{(1 + \beta )^{\frac{1}{n} + 1}} + \frac{1}{(1 - \beta )^{\frac{ -}{n} + 1}} \biggr]^{\frac{1}{q}} < \infty. \end{aligned} $$

By (12) and (13) (for $f = \tilde{f}_{n}$, $g = \tilde{g}_{n} $) we have

$$\begin{gathered} \frac{2}{1 - \beta^{2}} \int_{E_{ - \delta}} x_{\alpha}^{\delta (\sigma_{1} - \sigma + \frac{1}{n}) - 1}\,dx \int_{M_{\alpha,\beta}}^{\infty} e^{ - \rho u^{\gamma}} u^{\sigma - \frac{1}{qn} - 1}\,du \\ \quad \le J_{1} = \int_{ - \infty}^{\infty} \int_{ - \infty}^{\infty} e^{ - \rho (x_{\alpha}^{\delta} y_{\beta} )^{\gamma}} \tilde{f}_{n}(x) \tilde{g}_{n}(y)\,dx \,dy \le M\tilde{J}_{2} < \infty. \end{gathered} $$

Since for any $n \ge \frac{1}{\sigma - \sigma_{1}}$, $\sigma_{1} - \sigma + \frac{1}{n} \le 0 $, by Lemma 1 it follows that $\int_{E_{ - \delta}} x_{\alpha}^{\delta (\sigma_{1} - \sigma + \frac{1}{n}) - 1}\,dx = \infty $. In view of $\int_{M_{\alpha,\beta}}^{\infty} e^{ - \rho u^{\gamma}} u^{\sigma - \frac{1}{qn} - 1}\,du > 0 $, we have $\infty \le M\tilde{J}_{2} < \infty $, which is a contradiction.

Hence we conclude that $\sigma_{1} = \sigma $.

The lemma is proved. □

### Lemma 4

*If there exists a constant* *M* *such that*, *for any nonnegative measurable functions* $f(x) $ *and* $g(y) $ *in* $\mathbb{R} $,

14 $$ \begin{aligned}[b] & \int_{ - \infty}^{\infty} \int_{ - \infty}^{\infty} e^{ - \rho (x_{\alpha}^{\delta} y_{\beta} )^{\gamma}} f(x)g(y)\,dx \,dy \\ &\quad \le M \biggl[ \int_{ - \infty}^{\infty} x_{\alpha}^{p(1 - \delta \sigma ) - 1}f^{p}(x) \,dx \biggr]^{\frac{1}{p}} \biggl[ \int_{ - \infty}^{\infty} y_{\beta}^{q(1 - \sigma ) - 1}g^{q}(y) \,dy \biggr]^{\frac{1}{q}}, \end{aligned} $$

*then we have* $K_{\alpha,\beta}^{(\gamma )}(\sigma ) \le M $.

### Proof

For $\sigma_{1} = \sigma $, by (8) we have

$$\begin{gathered} I_{1} = \int_{E_{\delta}} I(x)x_{\alpha}^{\delta (\sigma - \frac{1}{pn}) - 1}\,dx = I_{1}^{( - )} + I_{1}^{( + )}, \\ I_{1}^{( - )} = \int_{E_{\delta}} I^{( - )}(x)x_{\alpha}^{\delta (\sigma - \frac{1}{pn}) - 1} \,dx, \qquad I_{1}^{( + )}: = \int_{E_{\delta}} I^{( + )}(x)x_{\alpha}^{\delta (\sigma - \frac{1}{pn}) - 1} \,dx. \end{gathered} $$

In view of the presented results, we find

15 $$\begin{aligned} I_{1}^{( - )} &= \frac{1}{1 - \beta} \int_{E_{\delta}} x_{\alpha}^{ - \frac{\delta}{n} - 1} \int_{0}^{(1 - \beta )x_{\alpha}^{\delta}} e^{ - \rho u^{\gamma}} u^{\sigma + \frac{1}{qn} - 1}\,du \,dx \\ &= \frac{1}{1 - \beta} \int_{E_{\delta}} x_{\alpha}^{ - \frac{\delta}{n} - 1} \biggl[ \int_{0}^{\infty} e^{ - \rho u^{\gamma}} u^{\sigma + \frac{1}{qn} - 1}\,du - \int_{(1 - \beta )x_{\alpha}^{\delta}}^{\infty} e^{ - \rho u^{\gamma}} u^{\sigma + \frac{1}{qn} - 1}\,du \biggr]\,dx \\ &= \frac{n}{1 - \beta} \biggl[ \frac{1}{(1 + \alpha )^{\frac{\delta}{n} + 1}} + \frac{1}{(1 - \alpha )^{\frac{\delta}{n} + 1}} \biggr]k_{\rho}^{(\gamma )}\biggl(\sigma + \frac{1}{qn}\biggr) \\ &\quad {}- \frac{1}{1 - \beta} \int_{E_{\delta}} x_{\alpha}^{ - \frac{\delta}{n} - 1} \int_{(1 - \beta )x_{\alpha}^{\delta}}^{\infty} e^{ - \rho u^{\gamma}} u^{\sigma + \frac{1}{qn} - 1}\,du \,dx. \end{aligned}$$

Since $e^{ - \rho u^{\gamma}} u^{2\sigma} $ is continuous in $(0,\infty ) $, and $e^{ - \rho u^{\gamma}} u^{2\sigma} \to 0$ ($u \to \infty $), there exists a positive constant $M_{1} $ such that $e^{ - \rho u^{\gamma}} u^{2\sigma} \le M_{1}$ ($u \in [m_{\alpha,\beta},\infty ) $). By (4) it follows that

$$\begin{aligned} 0 &< \int_{E_{\delta}} x_{\alpha}^{ - \frac{\delta}{n} - 1} \int_{(1 - \beta )x_{\alpha}^{\delta}}^{\infty} e^{ - \rho u^{\gamma}} u^{\sigma + \frac{1}{qn} - 1}\,du \,dx \\ & \le M_{1} \int_{E_{\delta}} x_{\alpha}^{ - \frac{\delta}{n} - 1}\biggl[ \int_{(1 - \beta )x_{\alpha}^{\delta}}^{\infty} u^{ - \sigma + \frac{1}{qn} - 1} \,du\biggr]\,dx = \frac{M_{1}\int_{E_{\delta}} x_{\alpha}^{ - \delta (\sigma + \frac{1}{pn}) - 1}\,dx}{(1 - \beta )^{\sigma - \frac{1}{qn}}} \\ &= \frac{(\sigma + \frac{1}{pn})^{ - 1}M_{1}}{(1 - \beta )^{\sigma - \frac{1}{qn}}} \biggl[ \frac{1}{(1 + \alpha )^{\delta (\sigma + \frac{1}{pn}) + 1}} + \frac{1}{(1 - \alpha )^{\delta (\sigma + \frac{1}{pn}) + 1}} \biggr], \end{aligned} $$

so that

$$\frac{1}{1 - \beta} \int_{E_{\delta}} x_{\alpha}^{ - \frac{\delta}{n} - 1} \int_{(1 - \beta )x_{\alpha}^{\delta}}^{\infty} e^{ - \rho u^{\gamma}} u^{\sigma + \frac{1}{qn} - 1}\,du \,dx = O(1). $$

By (15) it follows that

16 $$ \frac{1}{n}I_{1}^{( - )} = \frac{k_{\rho}^{(\gamma )}(\sigma + \frac{1}{qn})}{1 - \beta} \biggl[ \frac{1}{(1 + \alpha )^{\frac{\delta}{n} + 1}} + \frac{1}{(1 - \alpha )^{\frac{\delta}{n} + 1}} \biggr] - \frac{O(1)}{n}. $$

In the same way, we have

17 $$ \frac{1}{n}I_{1}^{( + )} = \frac{k_{\rho}^{(\gamma )}(\sigma + \frac{1}{qn})}{1 + \beta} \biggl[ \frac{1}{(1 + \alpha )^{\frac{\delta}{n} + 1}} + \frac{1}{(1 - \alpha )^{\frac{\delta}{n} + 1}} \biggr] - \frac{\tilde{O}(1)}{n}. $$

By (14) (for $f = f_{n}$, $g = g_{n} $), we have

$$\frac{1}{n}I_{1} = \frac{1}{n}\bigl(I_{1}^{( - )} + I_{1}^{( + )}\bigr) \le \frac{1}{n}M \tilde{J}_{1}. $$

For $n \to \infty $, by Fatou lemma (see [27]), (16), and (17) we find

$$\frac{2}{1 - \beta^{2}}\frac{2k_{\rho}^{(\gamma )}(\sigma )}{1 - \alpha^{2}} \le M\biggl(\frac{2}{1 - \alpha^{2}} \biggr)^{\frac{1}{p}}\biggl(\frac{2}{1 - \beta^{2}}\biggr)^{\frac{1}{q}}, $$

so that $K_{\alpha,\beta}^{(\gamma )}(\sigma ) = \frac{2k_{\rho}^{(\gamma )}(\sigma )}{(1 - \alpha^{2})^{1/q}(1 - \beta^{2})^{1/p}} \le M $.

The lemma is proved. □

### Lemma 5

*We define the following weight functions*:

18 $$\begin{aligned}& \omega_{\delta} (\sigma,y): = y_{\beta}^{\sigma} \int_{ - \infty}^{\infty} e^{ - \rho (x_{\alpha}^{\delta} y_{\beta} )^{\gamma}} x_{\alpha}^{\delta \sigma - 1} \,dx\quad (y \in \mathbb{R}), \end{aligned}$$

19 $$\begin{aligned}& \varpi_{\delta} (\sigma,x): = x_{\alpha}^{\delta \sigma} \int_{ - \infty}^{\infty} e^{ - \rho (x_{\alpha}^{\delta} y_{\beta} )^{\gamma}} y_{\beta}^{\sigma - 1} \,dy\quad (x \in \mathbb{R}). \end{aligned}$$

*Then we have*

20 $$ \frac{1 - \alpha^{2}}{2}\omega_{\delta} (\sigma,y) = \frac{1 - \beta^{2}}{2} \varpi_{\delta} (\sigma,x) = k_{\rho}^{(\gamma )}(\sigma )\quad \bigl(x,y \in \mathbb{R}\backslash \{ 0\} \bigr). $$

### Proof

For fixed $y \in \mathbb{R}\backslash \{ 0\} $, setting $u = x_{\alpha}^{\delta} y_{\beta} $, we find

$$\begin{aligned} \omega_{\delta} (\sigma,y) &= y_{\beta}^{\sigma} \int_{ - \infty}^{0} e^{ - \rho (x_{\alpha}^{\delta} y_{\beta} )^{\gamma}} \bigl[(1 - \alpha ) ( - x)\bigr]^{\delta \sigma - 1}\,dx \\ &\quad {}+ y_{\beta}^{\sigma} \int_{0}^{\infty} e^{ - \rho (x_{\alpha}^{\delta} y_{\beta} )^{\gamma}} \bigl[(1 + \alpha )x \bigr]^{\delta \sigma - 1}\,dx \\ &= \biggl(\frac{1}{1 - \alpha} + \frac{1}{1 + \alpha} \biggr) \int_{0}^{\infty} e^{ - \rho u^{\gamma}} u^{\sigma - 1}\,du = \frac{2}{1 - \alpha^{2}}k_{\rho}^{(\gamma )}(\sigma ); \end{aligned} $$

for fixed $x \in \mathbb{R}\backslash \{ 0\} $, setting $u = x_{\alpha}^{\delta} y_{\beta} $, it follows that

$$\begin{aligned} \varpi_{\delta} (\sigma,x) &= x_{\alpha}^{\delta \sigma} \int_{ - \infty}^{0} e^{ - \rho (x_{\alpha}^{\delta} y_{\beta} )^{\gamma}} y_{\beta}^{\sigma - 1}\,dy + x_{\alpha}^{\delta \sigma} \int_{0}^{\infty} e^{ - \rho (x_{\alpha}^{\delta} y_{\beta} )^{\gamma}} y_{\beta}^{\sigma - 1} \,dy \\ &= \frac{2}{1 - \beta^{2}} \int_{0}^{\infty} e^{ - \rho u^{\gamma}} u^{\sigma - 1}\,du = \frac{2}{1 - \beta^{2}}k_{\rho}^{(\gamma )}(\sigma ). \end{aligned} $$

Hence we have (20).

The lemma is proved. □

## Main results

### Theorem 1

*If* *M* *is a constant*, *then the following statements* (i), (ii), *and* (iii) *are equivalent*:

(i) *For any* $f(x) \ge 0 $, *we have*:
  21 $$ \begin{aligned}[b] J&: = \biggl\{ \int_{ - \infty}^{\infty} y_{\beta}^{p\sigma - 1} \biggl[ \int_{ - \infty}^{\infty} e^{ - \rho (x_{\alpha}^{\delta} y_{\beta} )^{\gamma}} f(x)\,dx \biggr]^{p}\,dy \biggr\} ^{\frac{1}{p}} \\ &\le M \biggl[ \int_{ - \infty}^{\infty} x_{\alpha}^{p(1 - \delta \sigma_{1}) - 1}f^{p}(x) \,dx \biggr]^{\frac{1}{p}}. \end{aligned} $$
(ii) *For any* $f(x),g(y) \ge 0 $, *we have*:
  22 $$ \begin{aligned}[b] I &= \int_{ - \infty}^{\infty} \int_{ - \infty}^{\infty} e^{ - \rho (x_{\alpha}^{\delta} y_{\beta} )^{\gamma}} f(x)g(y)\,dx \,dy \\ &\le M \biggl[ \int_{ - \infty}^{\infty} x_{\alpha}^{p(1 - \delta \sigma_{1}) - 1}f^{p}(x) \,dx \biggr]^{\frac{1}{p}} \biggl[ \int_{ - \infty}^{\infty} y_{\beta}^{q(1 - \sigma ) - 1}g^{q}(y) \,dy \biggr]^{\frac{1}{q}}. \end{aligned} $$
(iii) $\sigma_{1} = \sigma $, *and* $K_{\alpha,\beta}^{(\gamma )}(\sigma ) \le M $.

### Proof

(i)=>(ii). By Hölder’s inequality (see [28]) we have

23 $$ \begin{aligned}[b] I &= \int_{ - \infty}^{\infty} \biggl[ y_{\beta}^{\sigma - \frac{1}{p}} \int_{ - \infty}^{\infty} e^{ - \rho (x_{\alpha}^{\delta} y_{\beta} )^{\gamma}} f(x)\,dx \biggr] \bigl( y_{\beta}^{ - \sigma + \frac{1}{p}}g(y) \bigr)\,dy \\ &\le J \biggl[ \int_{ - \infty}^{\infty} y_{\beta}^{q(1 - \sigma ) - 1}g^{q}(y) \,dy \biggr]^{\frac{1}{q}}. \end{aligned} $$

Then by (21) we have (22).

(ii)=>(iii). By Lemma 1 we have $\sigma_{1} = \sigma $. Then by Lemma 2 we have $K_{\alpha,\beta}^{(\gamma )}(\sigma ) \le M $.

(iii)=>(i). For $\sigma_{1} = \sigma $, by Hölder’s inequality with weight (see [28]) and (18) we have

24 $$ \begin{gathered}[b] \biggl[ \int_{ - \infty}^{\infty} e^{ - \rho (x_{\alpha}^{\delta} y_{\beta} )^{\gamma}} f(x)\,dx \biggr]^{p} \\ \quad = \biggl\{ \int_{ - \infty}^{\infty} e^{ - \rho (x_{\alpha}^{\delta} y_{\beta} )^{\gamma}} \biggl[ \frac{y_{\beta}^{(\sigma - 1)/p}f(x)}{x_{\alpha}^{(\delta \sigma - 1)/q}} \biggr] \biggl[ \frac{x_{\alpha}^{(\delta \sigma - 1)/q}}{y_{\beta}^{(\sigma - 1)/p}} \biggr]\,dx \biggr\} ^{p} \\ \quad \le \int_{ - \infty}^{\infty} e^{ - \rho (x_{\alpha}^{\delta} y_{\beta} )^{\gamma}} \frac{y_{\beta}^{\sigma - 1}f^{p}(x)}{x_{\alpha}^{(\delta \sigma - 1)p/q}} \,dx \biggl[ \int_{ - \infty}^{\infty} e^{ - \rho (x_{\alpha}^{\delta} y_{\beta} )^{\gamma}} \frac{x_{\alpha}^{\delta \sigma - 1}}{y_{\beta}^{(\sigma - 1)q/p}} \,dx \biggr]^{p/q} \\ \quad = \bigl[\omega_{\delta} (\sigma,y)y_{\beta}^{q(1 - \sigma ) - 1} \bigr]^{p - 1} \int_{ - \infty}^{\infty} e^{ - \rho (x_{\alpha}^{\delta} y_{\beta} )^{\gamma}} \frac{y_{\beta}^{\sigma - 1}f^{p}(x)}{x_{\alpha}^{(\delta \sigma - 1)p/q}} \,dx \\ \quad = \biggl( \frac{2k_{\rho}^{(\gamma )}(\sigma )}{1 - \alpha^{2}} \biggr)^{p - 1}y_{\beta}^{ - p\sigma + 1} \int_{ - \infty}^{\infty} e^{ - \rho (x_{\alpha}^{\delta} y_{\beta} )^{\gamma}} \frac{y_{\beta}^{\sigma - 1}f^{p}(x)}{x_{\alpha}^{(\delta \sigma - 1)p/q}} \,dx. \end{gathered} $$

By Fubini’s theorem (see [27]), (24), and (19) we have

$$\begin{aligned} J &\le \biggl( \frac{2k_{\rho}^{(\gamma )}(\sigma )}{1 - \alpha^{2}} \biggr)^{\frac{1}{q}} \biggl[ \int_{ - \infty}^{\infty} \int_{ - \infty}^{\infty} e^{ - \rho (x_{\alpha}^{\delta} y_{\beta} )^{\gamma}} \frac{y_{\beta}^{\sigma - 1}f^{p}(x)}{x_{\alpha}^{(\delta \sigma - 1)p/q}} \,dx \,dy \biggr]^{\frac{1}{p}} \\ &= \biggl( \frac{2k_{\rho}^{(\gamma )}(\sigma )}{1 - \alpha^{2}} \biggr)^{\frac{1}{q}} \biggl[ \int_{ - \infty}^{\infty} \varpi_{\delta} ( \sigma,x)x_{\delta}^{p(1 - \delta \sigma ) - 1}f^{p}(x)\,dx \biggr]^{\frac{1}{p}} \\ &= K_{\alpha,\beta}^{(\gamma )}(\sigma ) \biggl[ \int_{ - \infty}^{\infty} x_{\delta}^{p(1 - \delta \sigma ) - 1}f^{p}(x) \,dx \biggr]^{\frac{1}{p}}. \end{aligned} $$

For $K_{\alpha,\beta}^{(\gamma )}(\sigma ) \le M $, we have (21) (when $\sigma_{1} = \sigma $).

Therefore, statements (i), (ii), and (iii) are equivalent.

The theorem is proved. □

### Theorem 2

*If* *M* *is a constant*, *then the following statements* (i), (ii), *and* (iii) *are equivalent*:

(i) *For any* $f ( x ) \geq 0$ *satisfying* $0 < \int_{ - \infty}^{\infty} x_{\alpha}^{p(1 - \delta \sigma ) - 1}f^{p}(x)\,dx < \infty $, *we have*:
  25 $$ \begin{gathered}[b] \biggl\{ \int_{ - \infty}^{\infty} y_{\beta}^{p\sigma - 1} \biggl[ \int_{ - \infty}^{\infty} e^{ - \rho (x_{\alpha}^{\delta} y_{\beta} )^{\gamma}} f(x)\,dx \biggr]^{p}\,dy \biggr\} ^{\frac{1}{p}} \\ \quad < M \biggl[ \int_{ - \infty}^{\infty} x_{\alpha}^{p(1 - \delta \sigma ) - 1}f^{p}(x) \,dx \biggr]^{\frac{1}{p}}. \end{gathered} $$
(ii) *For any* $f ( x ) \geq 0$ *satisfying* $0 < \int_{ - \infty}^{\infty} x_{\alpha}^{p(1 - \delta \sigma ) - 1}f^{p}(x)\,dx < \infty $, *and* $g ( x ) \geq 0$ *satisfying* $0 < \int_{ - \infty}^{\infty} y_{\beta}^{q(1 - \sigma ) - 1}g^{q}(y)\,dy < \infty $, *we have*:
  26 $$ \begin{gathered}[b] \int_{ - \infty}^{\infty} \int_{ - \infty}^{\infty} e^{ - \rho (x_{\alpha}^{\delta} y_{\beta} )^{\gamma}} f(x)g(y)\,dx \,dy \\ \quad < M \biggl[ \int_{ - \infty}^{\infty} x_{\alpha}^{p(1 - \delta \sigma ) - 1}f^{p}(x) \,dx \biggr]^{\frac{1}{p}} \biggl[ \int_{ - \infty}^{\infty} y_{\beta}^{q(1 - \sigma ) - 1}g^{q}(y) \,dy \biggr]^{\frac{1}{q}}. \end{gathered} $$
(iii) $K_{\alpha,\beta}^{(\gamma )}(\sigma ) \le M $.

*Moreover*, *if statement* (iii) *holds*, *then the constant factor* $M = K_{\alpha,\beta}^{(\gamma )}(\sigma ) $ *in* (25) *and* (26) *is the best possible*.

*In particular*, (1) *for* $\delta = 1$, $M = K_{\alpha,\beta}^{(\gamma )}(\sigma ) $, *we have the following equivalent inequalities with nonhomogeneous kernel*:

27 $$\begin{aligned}& \begin{gathered}[b] \biggl\{ \int_{ - \infty}^{\infty} y_{\beta}^{p\sigma - 1} \biggl[ \int_{ - \infty}^{\infty} e^{ - \rho (x_{\alpha} y_{\beta} )^{\gamma}} f(x)\,dx \biggr]^{p}\,dy \biggr\} ^{\frac{1}{p}} \\ \quad < K_{\alpha,\beta}^{(\gamma )}(\sigma ) \biggl[ \int_{ - \infty}^{\infty} x_{\alpha}^{p(1 - \sigma ) - 1}f^{p}(x) \,dx \biggr]^{\frac{1}{p}}, \end{gathered} \end{aligned}$$

28 $$\begin{aligned}& \begin{gathered}[b] \int_{ - \infty}^{\infty} \int_{ - \infty}^{\infty} e^{ - \rho (x_{\alpha} y_{\beta} )^{\gamma}} f(x)g(y)\,dx \,dy \\ \quad < K_{\alpha,\beta}^{(\gamma )}(\sigma ) \biggl[ \int_{ - \infty}^{\infty} x_{\alpha}^{p(1 - \sigma ) - 1}f^{p}(x) \,dx \biggr]^{\frac{1}{p}} \biggl[ \int_{ - \infty}^{\infty} y_{\beta}^{q(1 - \sigma ) - 1}g^{q}(y) \,dy \biggr]^{\frac{1}{q}}, \end{gathered} \end{aligned}$$

*where* $K_{\alpha,\beta}^{(\gamma )}(\sigma ) $ *is the best possible constant factor*;

(2) *for* $\delta = - 1$, $M = K_{\alpha,\beta}^{(\gamma )}(\sigma ) $, *we have the following equivalent inequalities with homogeneous kernel of degree* 0:

29 $$\begin{aligned}& \begin{gathered}[b] \biggl\{ \int_{ - \infty}^{\infty} y_{\beta}^{p\sigma - 1} \biggl[ \int_{ - \infty}^{\infty} e^{ - \rho (y_{\beta} /x_{\alpha} )^{\gamma}} f(x)\,dx \biggr]^{p}\,dy \biggr\} ^{\frac{1}{p}} \\ \quad < K_{\alpha,\beta}^{(\gamma )}(\sigma ) \biggl[ \int_{ - \infty}^{\infty} x_{\alpha}^{p(1 + \sigma ) - 1}f^{p}(x) \,dx \biggr]^{\frac{1}{p}}, \end{gathered} \end{aligned}$$

30 $$\begin{aligned}& \begin{gathered}[b] \int_{ - \infty}^{\infty} \int_{ - \infty}^{\infty} e^{ - \rho (y_{\beta} /x_{\alpha} )^{\gamma}} f(x)g(y)\,dx \,dy \\ \quad < K_{\alpha,\beta}^{(\gamma )}(\sigma ) \biggl[ \int_{ - \infty}^{\infty} x_{\alpha}^{p(1 + \sigma ) - 1}f^{p}(x) \,dx \biggr]^{\frac{1}{p}} \biggl[ \int_{ - \infty}^{\infty} y_{\beta}^{q(1 - \sigma ) - 1}g^{q}(y) \,dy \biggr]^{\frac{1}{q}}, \end{gathered} \end{aligned}$$

*where* $K_{\alpha,\beta}^{(\gamma )}(\sigma ) $ *is the best possible constant factor*.

### Proof

For $\sigma_{1} = \sigma $, under and the assumption of statement (i), if (24) takes the form of equality for $y \in \mathbb{R}\backslash \{ 0\} $, then there exist constants *A* and *B* such that they are not both zero and (see [28])

$$A\frac{y_{\beta}^{\sigma - 1}}{x_{\alpha}^{(\delta \sigma - 1)p/q}}f^{p}(x) = B\frac{x_{\alpha}^{\delta \sigma - 1}}{y_{\beta}^{(\sigma - 1)q/p}}\quad \mbox{a.e. in }\mathbb{R}. $$

We suppose that $A \ne 0 $ (otherwise, $B = A = 0 $). Then it follows that

$$x_{\alpha}^{p(1 - \delta \sigma ) - 1}f^{p}(x) = y_{\beta}^{q(\sigma - 1)} \frac{B}{Ax_{\alpha}}\quad \mbox{a.e. in }\mathbb{R}. $$

Since $\int_{ - \infty}^{\infty} x_{\alpha}^{ - 1} \,dx = \infty $, this contradicts the fact that $0 < \int_{ - \infty}^{\infty} x_{\alpha}^{p(1 - \delta \sigma ) - 1} f^{p}(x)\,dx < \infty $. Hence (24) takes the form of strict inequality, and so does (21). Hence (25) and (26) are valid.

In view of Theorem 1, we still can conclude that statements (i), (ii), and (iii) in Theorem 2 are equivalent.

When statement (iii) holds, namely, $K_{\alpha,\beta}^{(\gamma )}(\sigma ) \le M $, if there exists a constant $M( \le K_{\alpha,\beta}^{(\gamma )}(\sigma )) $ such that (26) is valid, then $M = K_{\alpha,\beta}^{(\gamma )}(\sigma ) $, and we can conclude that the constant factor $M = K_{\alpha,\beta}^{(\gamma )}(\sigma ) $ in (26) is the best possible.

The constant factor $M = K_{\alpha,\beta}^{(\gamma )}(\sigma ) $ in (25) is still the best possible. Otherwise, by (23) (for $\sigma_{1} = \sigma $), we would get a contradiction that the constant factor $M = K_{\alpha,\beta}^{(\gamma )}(\sigma ) $ in (26) is not the best possible.

The theorem is proved. □

## Operator expressions

We set the following functions: $\varphi (x): = x_{\alpha}^{p(1 - \delta \sigma ) - 1}$ ($x \in \mathbb{R} $), and $\psi (y): = y_{\beta}^{q(1 - \sigma ) - 1} $, where from $\psi^{1 - p}(y): = y_{\beta}^{p\sigma - 1}$ ($y \in \mathbb{R} $). Define the following real normed linear spaces:

$$\begin{gathered} L_{p,\varphi} (\mathbb{R}): = \biggl\{ f; \Vert f \Vert _{p,\varphi}: = \biggl( \int_{ - \infty}^{\infty} \varphi (x) \bigl\vert f(x) \bigr\vert ^{p}\,dx \biggr)^{\frac{1}{p}} < \infty \biggr\} , \\ L_{q,\psi} (\mathbb{R}): = \biggl\{ g; \Vert g \Vert _{q,\psi} = \biggl( \int_{ - \infty}^{\infty} \psi (y) \bigl\vert g(y) \bigr\vert ^{q}\,dy \biggr)^{\frac{1}{q}} < \infty \biggr\} , \\ L_{p,\psi^{1 - p}}(\mathbb{R}): = \biggl\{ h; \Vert h \Vert _{p,\psi^{1 - p}} = \biggl( \int_{ - \infty}^{\infty} \psi^{1 - p}(y) \bigl\vert h(y) \bigr\vert ^{p}\,dy \biggr)^{\frac{1}{p}} < \infty \biggr\} . \end{gathered} $$

In view of Theorem 2, for $f \in L_{p,\varphi} (\mathbb{R}) $, setting

$$h_{1}(y): = \int_{ - \infty}^{\infty} e^{ - \rho (y_{\beta} /x_{\alpha} )^{\gamma}} f(x)\,dx \quad (y \in \mathbb{R}), $$

by (25) we have

31 $$ \Vert h_{1} \Vert _{p,\psi^{1 - p}} = \biggl( \int_{ - \infty}^{\infty} \psi^{1 - p}(y) \bigl\vert h_{1}(y) \bigr\vert ^{p}\,dy \biggr)^{\frac{1}{p}} \le M \Vert f \Vert _{p,\varphi} < \infty. $$

### Definition 1

Define the Hilbert-type integral operator $T:L_{p,\varphi} (\mathbb{R}) \to L_{p,\psi^{1 - p}}(\mathbb{R}) $ as follows: For any $f \in L_{p,\varphi} (\mathbb{R}) $, there exists a unique representation $Tf = h_{1} \in L_{p,\psi^{1 - p}}(\mathbb{R}) $, satisfying for any $y \in \mathbb{R}$, $Tf(y) = h_{1}(y) $.

In view of (31), it follows that $\Vert Tf\Vert _{p,\psi^{1 - p}} = \Vert h_{1}\Vert _{p,\psi^{1 - p}} \le M\Vert f\Vert _{p,\varphi} $, and then the operator *T* is bounded and satisfies

$$\Vert T \Vert = \sup_{f( \ne \theta ) \in L_{p,\varphi} (\mathbb{R})}\frac{ \Vert Tf \Vert _{p,\psi^{1 - p}}}{ \Vert f \Vert _{p,\varphi}} \le M. $$

If we define the formal inner product of *Tf* and *g* as

$$(Tf,g): = \int_{ - \infty}^{\infty} \biggl[ \int_{ - \infty}^{\infty} e^{ - \rho (y_{\beta} /x_{\alpha} )^{\gamma}} f(x)\,dx\biggr]g(y) \,dy, $$

then we can rewrite Theorem 2 as follows.

### Theorem 3

*If* *M* *is a constant*, *then the following statements* (i), (ii), *and* (iii) *are equivalent*:

(i) *For any* $f(x) \ge 0$, $f \in L_{p,\varphi} (\mathbb{R})$, $\Vert f\Vert _{p,\varphi} > 0 $, *we have*:
  32 $$ \Vert Tf \Vert _{p,\psi^{1 - p}} < M \Vert f \Vert _{p,\varphi}. $$
(ii) *For* $f(x),g(y) \ge 0$, $f \in L_{p,\varphi} (\mathbb{R})$, $g \in L_{q,\psi} (\mathbb{R}) $, $\Vert f\Vert _{p,\varphi},\Vert g\Vert _{q,\psi} > 0 $, *we have*:
  33 $$ (Tf,g) < M \Vert f \Vert _{p,\varphi} \Vert g \Vert _{q,\psi}. $$
(iii) $K_{\alpha,\beta}^{(\gamma )}(\sigma ) \le M $.

*Moreover*, *if statement* (iii) *holds*, *then the constant factor* $M = K_{\alpha,\beta}^{(\gamma )}(\sigma ) $ *in* (32) *and* (33) *is the best possible*, *namely*, $\Vert T\Vert = K_{\alpha,\beta}^{(\gamma )}(\sigma ) $.

### Remark 1

(1) In particular, for $\alpha = \beta = 0 $ in (27) and (28), we have the following equivalent inequalities:

34 $$\begin{aligned}& \begin{gathered}[b] \biggl\{ \int_{ - \infty}^{\infty} \vert y \vert ^{p\sigma - 1} \biggl[ \int_{ - \infty}^{\infty} e^{ - \rho \vert xy \vert ^{\gamma}} f(x)\,dx \biggr]^{p}\,dy \biggr\} ^{\frac{1}{p}} \\ \quad < \frac{2\Gamma (\sigma /\gamma )}{\gamma \rho^{\sigma /\gamma}} \biggl[ \int_{ - \infty}^{\infty} \vert x \vert ^{p(1 - \sigma ) - 1}f^{p}(x) \,dx \biggr]^{\frac{1}{p}}, \end{gathered} \end{aligned}$$

35 $$\begin{aligned}& \begin{gathered}[b] \int_{ - \infty}^{\infty} \int_{ - \infty}^{\infty} e^{ - \rho \vert xy \vert ^{\gamma}} f(x)g(y)\,dx \,dy \\ \quad < \frac{2\Gamma (\sigma /\gamma )}{\gamma \rho^{\sigma /\gamma}} \biggl[ \int_{ - \infty}^{\infty} \vert x \vert ^{p(1 - \sigma ) - 1}f^{p}(x) \,dx \biggr]^{\frac{1}{p}} \biggl[ \int_{ - \infty}^{\infty} \vert y \vert ^{q(1 - \sigma ) - 1}g^{q}(y) \,dy \biggr]^{\frac{1}{q}}, \end{gathered} \end{aligned}$$

where $\frac{2\Gamma (\sigma /\gamma )}{\gamma \rho^{\sigma /\gamma}} $ is the best possible constant factor.

If $f( - x) = f(x)$, $g( - y) = g(y)$ ($x,y \in \mathbb{R}_{ +} $), then we have the following equivalent inequalities:

36 $$\begin{aligned}& \begin{gathered}[b] \biggl\{ \int_{0}^{\infty} y^{p\sigma - 1} \biggl[ \int_{0}^{\infty} e^{ - \rho (xy)^{\gamma}} f(x)\,dx \biggr]^{p}\,dy \biggr\} ^{\frac{1}{p}} \\ \quad < \frac{\Gamma (\sigma /\gamma )}{\gamma \rho^{\sigma /\gamma}} \biggl[ \int_{0}^{\infty} x^{p(1 - \sigma ) - 1}f^{p}(x) \,dx \biggr]^{\frac{1}{p}}, \end{gathered} \end{aligned}$$

37 $$\begin{aligned}& \begin{gathered}[b] \int_{0}^{\infty} \int_{0}^{\infty} e^{ - \rho (xy)^{\gamma}} f(x)g(y)\,dx \,dy \\ \quad < \frac{\Gamma (\sigma /\gamma )}{\gamma \rho^{\sigma /\gamma}} \biggl[ \int_{0}^{\infty} x^{p(1 - \sigma ) - 1}f^{p}(x) \,dx \biggr]^{\frac{1}{p}} \biggl[ \int_{0}^{\infty} y^{q(1 - \sigma ) - 1}g^{q}(y) \,dy \biggr]^{\frac{1}{q}}, \end{gathered} \end{aligned}$$

where $\frac{\Gamma (\sigma /\gamma )}{\gamma \rho^{\sigma /\gamma}} $ is the best possible constant factor.

(2) For $\alpha = \beta = 0 $ in (29) and (30), we have the following equivalent inequalities:

38 $$\begin{aligned}& \begin{gathered}[b] \biggl\{ \int_{ - \infty}^{\infty} \vert y \vert ^{p\sigma - 1} \biggl[ \int_{ - \infty}^{\infty} e^{ - \rho \vert y/x \vert ^{\gamma}} f(x)\,dx \biggr]^{p}\,dy \biggr\} ^{\frac{1}{p}} \\ \quad < \frac{2\Gamma (\sigma /\gamma )}{\gamma \rho^{\sigma /\gamma}} \biggl[ \int_{ - \infty}^{\infty} \vert x \vert ^{p(1 + \sigma ) - 1}f^{p}(x) \,dx \biggr]^{\frac{1}{p}}, \end{gathered} \end{aligned}$$

39 $$\begin{aligned}& \begin{gathered}[b] \int_{ - \infty}^{\infty} \int_{ - \infty}^{\infty} e^{ - \rho \vert y/x \vert ^{\gamma}} f(x)g(y)\,dx \,dy \\ \quad < \frac{2\Gamma (\sigma /\gamma )}{\gamma \rho^{\sigma /\gamma}} \biggl[ \int_{ - \infty}^{\infty} \vert x \vert ^{p(1 + \sigma ) - 1}f^{p}(x) \,dx \biggr]^{\frac{1}{p}} \biggl[ \int_{ - \infty}^{\infty} \vert y \vert ^{q(1 - \sigma ) - 1}g^{q}(y) \,dy \biggr]^{\frac{1}{q}}, \end{gathered} \end{aligned}$$

where $\frac{2\Gamma (\sigma /\gamma )}{\gamma \rho^{\sigma /\gamma}} $ is the best possible constant factor.

If $f( - x) = f(x)$, $g( - y) = g(y)$ ($x,y \in \mathbb{R}_{ +} $), then we have the following equivalent inequalities:

40 $$\begin{aligned}& \begin{gathered}[b] \biggl\{ \int_{0}^{\infty} y^{p\sigma - 1} \biggl[ \int_{0}^{\infty} e^{ - \rho (y/x)^{\gamma}} f(x)\,dx \biggr]^{p}\,dy \biggr\} ^{\frac{1}{p}} \\ \quad < \frac{\Gamma (\sigma /\gamma )}{\gamma \rho^{\sigma /\gamma}} \biggl[ \int_{0}^{\infty} x^{p(1 + \sigma ) - 1}f^{p}(x) \,dx \biggr]^{\frac{1}{p}}, \end{gathered} \end{aligned}$$

41 $$\begin{aligned}& \begin{gathered}[b] \int_{0}^{\infty} \int_{0}^{\infty} e^{ - \rho (y/x)^{\gamma}} f(x)g(y)\,dx \,dy \\ \quad < \frac{\Gamma (\sigma /\gamma )}{\gamma \rho^{\sigma /\gamma}} \biggl[ \int_{0}^{\infty} x^{p(1 + \sigma ) - 1}f^{p}(x) \,dx \biggr]^{\frac{1}{p}} \biggl[ \int_{0}^{\infty} y^{q(1 - \sigma ) - 1}g^{q}(y) \,dy \biggr]^{\frac{1}{q}}, \end{gathered} \end{aligned}$$

where $\frac{\Gamma (\sigma /\gamma )}{\gamma \rho^{\sigma /\gamma}} $ is the best possible constant factor.

## Conclusions

In this paper, by using real analysis and weight functions we obtain a few equivalent statements of a Hilbert-type integral inequality in the whole plane related to the kernel of exponent function with the intermediate variables (Theorem 1). The constant factor related to the gamma function is proved to be the best possible in Theorem 2. We also consider some particular cases and the operator expressions in Remark 1 and Theorem 3. The lemmas and theorems provide an extensive account of this type of inequalities.
